# In Memory of Michael G. Rossmann: A Wise Man with a Forever Young Heart

**DOI:** 10.3390/v13071305

**Published:** 2021-07-05

**Authors:** Chuan (River) Xiao

**Affiliations:** Department of Chemistry and Biochemistry, University of Texas at El Paso, El Paso, TX 79968, USA; cxiao@utep.edu

Whenever I think about Michael’s passing, a sad feeling still strikes me. Two months before his eighty-ninth birthday, Michael left us and his beloved scientific research. His legendary achievements have been reviewed in several memorial articles [1,2,3]. Thus, I will not repeat here what has already been well written. Instead, I want to remember Michael as a great human being, whose impact touched many.

Before I met Michael, I read his seminal publication on the glyceraldehyde 3-phosphate dehydrogenase (GAPDH) structure. I used that structure as a template to model Rice GAPDH, which I manually sequenced in China. Therefore, I felt so lucky when I joined Michael’s group in 1998. I still remember the very first thing Michael taught me: “do not call me Professor Rossmann; call me Michael.” He described the historical association with the title “Professor” in the UK, stating that he would rather earn respect from his knowledge not his title. In the introductory lecture to my large undergraduate biochemistry classes, I always relay this story and tell my students that they can call me by my first name, River. This is just one example of how Michael influenced the culture at Purdue, where it is common for students to address professors by their first names, which is not the normal practice at many other places.

Once, on my way to a regional tourist site after a seminar talk at El Paso, I was stopped by the Border Patrol Police. Since I was traveling inside the US, I did not carry my passport. I was almost deported back to China. After hearing this story, Michael was livid and insisted on writing to his US Senator. In his letter, he described his childhood in Nazi Germany where all Jewish people were mandated to wear the Star of David. He believed that the US was becoming a police state by requiring immigrants to carry passports at all times. Obviously, Michael had a very difficult childhood in Nazi Germany [4]. For example, his math teacher beat him on the face with a ruler; his classmates bullied him on his way home. Thus, every day he needed to devise a clever escape route. By contrast, he often praised his German teacher who escorted him to school every morning to protect him. Considering his past, it is easy to understand why Michael wanted to be a kind teacher, close to and caring for his mentees. After seeing the evil side of discrimination, Michael embraced diversity and had colleagues, friends, mentees, and collaborators from a wide variety of cultural and ethnic backgrounds.

Each of us have our own memory of Michael. Thus, before writing this article, I asked these questions: how can I best describe Michael’s personality? And which characteristics were his most important ones? My answers: Michael had a childlike heart that shined through his personality, even during his last days. It was so pure and innocent, full of curiosity and energy. It was manifested in his exceptional wisdom and sometimes intimidating seriousness when discussing science.

Michael loved skiing, a sport he learned from his postdoctoral mentor Max Perutz (Figure 1). I was lucky to learn skiing from Michael. In front of snow-covered slopes, Michael was a different person. He was always eager to “just go” regardless of the trail’s difficulty level. Once, before taking the chairlift, we agreed that we would first study the trail map before we went down. However, when I got to the top, Michael was nowhere to be found! He had no patience, just like a child longing for the things he wanted. Later, I found him on a mogul-filled slope, unable to stand up and was very close to a big “Avalanche Area” sign. Once again, his eagerness and competitive nature put Michael in a precarious situation. At meals during many ski trips, we would hear Michael complain, “I taught my children and students how to ski and now everyone is better than me.” He never considered his age to be a contributing factor. Until his health prevented it, Michael was skiing until the age of 82 [1]. Recently, I saw a picture of Michael, trying a hoverboard, like a kid. It was so ‘Michael’ (Figure 2), always wanting to try new things, here trying to recapture his joy of skiing on the hoverboard.

This childlike eagerness and impatience to adventure is reflected in his beloved science and research. We all have stories of Michael asking for experiments and calculations to carry out, not tomorrow but today, and maybe even yesterday! Perhaps this explains why Michael pursued protein crystallography when the field could only solve organic compounds, or his courage to pioneer virus structure determination when most considered it a crazy endeavor. The enthusiasm behind Michael’s young heart never diminished, which has influenced many of us to dive into “dangerous” slopes with him [5].

Another of Michael’s long-time hobbies was sailing, a sport about which I knew nothing until I became his crew for almost ten years. When sailing, his childlike desire to win was always apparent, especially at the finish line. However, unlike a child, he understood that winning required planning and hard work. We spent hours cleaning his sailboat, checking the lines and the knots. Ironically, in our eagerness to win, our boat had capsized. I felt I was drowning and climbed onto a rescue boat. When I looked back, Michael was still in water, dragging the boat to the shore. When Michael set his mind on something, he did not give up, and strived to insure everything was perfect, both in sailing and in science. I remember generating a figure for my first publication, Michael pulled out a ruler and found that there was a small error, something was off by less than 1 mm. He said, “redo the figure.” Computer software was not as sophisticated as it is today. Thus, remaking the figure was a time consuming and laborious endeavor. Just as a sailing race: as we approached the finish line, Michael would not tolerate any mistakes, no matter how tiny, to the line and knot settings. His strong desire to win, requisite and persistent hard work, and perfectionism also showed in his research, which led to many breakthroughs.

After Michael’s passing, I began to collect pictures from mentees, colleagues, and friends. Michael always has a very attractive and genuine smile (Figure 1 and Figure 2). However, I think the happiest smiles are in the pictures of Michael looking at structures (Figure 3). I could feel his joy and the happiness of a child playing with his most-loved toys or when his Christmas wishes were fulfilled. For Michael, the fulfilment of his curiosity made him the happiest. Although his curiosity was strongest for science, it extended to different cultures, history, the universe, and philosophy. His scientific curiosity was pure without utilitarianism. Once, Michael compared his group to another famous structural virology group. He noted that his group was unique in having bacteriophage projects. Michael liked to work on “cold” (unpopular) projects and make them “hot” (popular). In a pragmatism-dominated modern society, it is hard to find an innocent child seeking smoother pebbles or prettier shells at the shore of the scientific ocean.

Michael did not want to leave us or his beloved science. In his last days, he was still planning “post-recovery” research. Perhaps, he is still doing research in another dimension. When I teach about the Rossmann fold, I hear him saying, “call it the nucleotide binding motif. The name should inform its function not who solved the structure.” In closing, I would like to quote one of my students, “It is our responsibility and honor to carry on Michael’s legacy and keep him alive in our memory.” I think this special issue well serves this purpose.

## Figures and Tables

**Figure 1:**
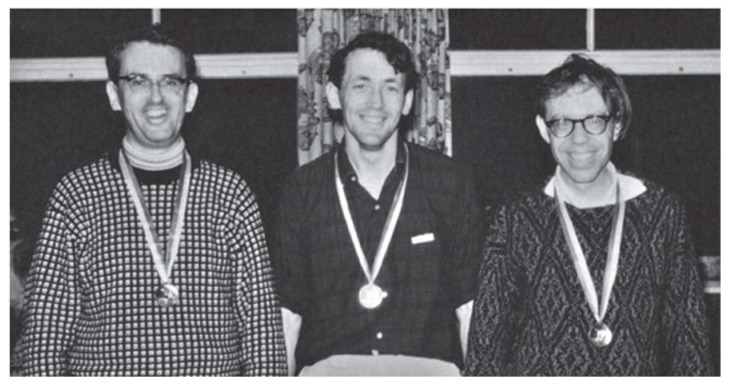
Michael won a bronze medal in a group beginner’s skiing competition at Hirschegg in 1966 [6].

**Figure 2:**
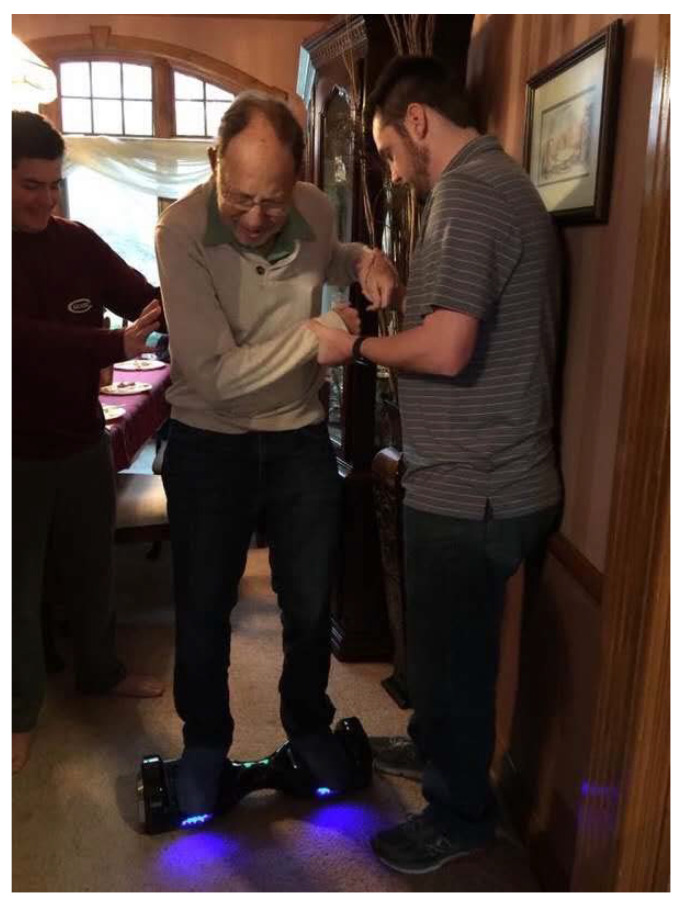
Michael was trying Hoverboard on 24 December 2017 (Photograph courtesy of Karen Bogan).

**Figure 3:**
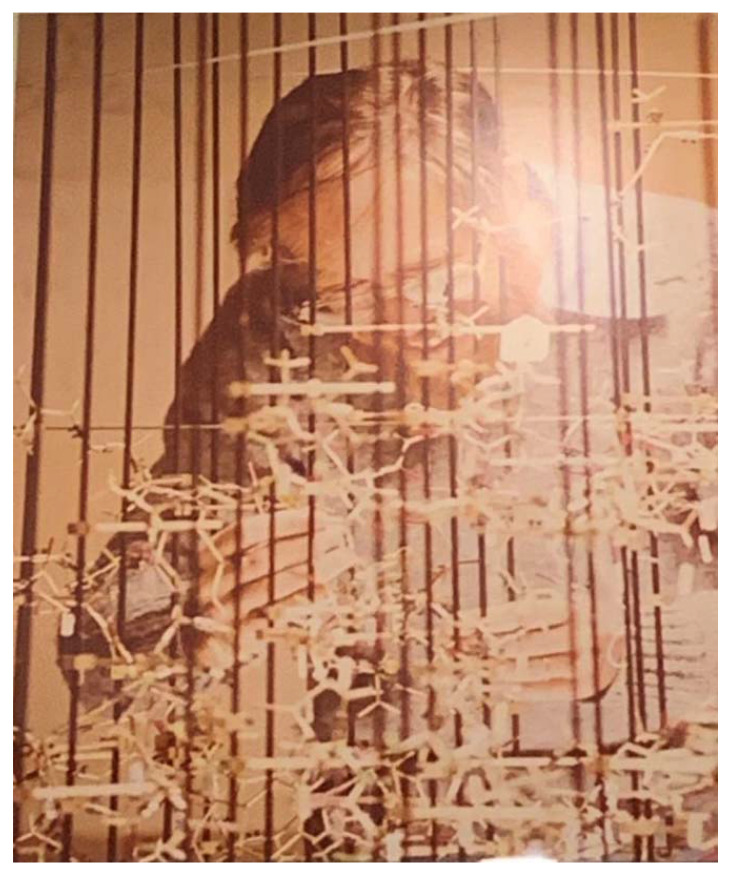
Michael’s smile when looking at structures.
